# Northampton General Hospital

**Published:** 1914-03-14

**Authors:** Fred Smith

**Affiliations:** House Governor. The Warneford, Leamington, and South Warwickshire General Hospital, Leamington


*To the Editor of* The Hospital.

### Northampton General Hospital.

*To the Editor of* The Hospital.

Sir,—Referring to the above and your comments in your issue of March 7, 1914 (p. 617), I should be obliged if you would correct the statement with regard to the pay of sisters at this hospital.

The salaries of our sisters commence at £30, and rise by annual increments of £2 10s. to £40. We have at present only one sister on our staff receiving the minimum salary of £30. In addition, the committee make a contribution towards the premiums of any who are members of the Royal Nurses' Pension Fund.—Yours faithfully,

Fred Smith, House Governor.

The Warneford, Leamington, and South Warwickshire
General Hospital, Leamington,
March 9, 1914.

[The statement to which Mr. Smith refers, that "in the neighbouring hospital of Leamington the sisters' salaries commence at £25, rising to £35," was not made by The Hospital, but by Mr. Manfield, the chairman of the Northampton General Hospital, in the course of his letter to the Editor.—Ed. The Hospital.]

